# A New Dictionary Construction Based Multimodal Medical Image Fusion Framework

**DOI:** 10.3390/e21030267

**Published:** 2019-03-09

**Authors:** Fuqiang Zhou, Xiaosong Li, Mingxuan Zhou, Yuanze Chen, Haishu Tan

**Affiliations:** 1 School of Instrumentation and Optoelectronic Engineering, Beihang University, Beijing 100083, China; 2 School of Physics and Optoelectronic Engineering, Foshan University, Foshan 528000, China

**Keywords:** medical image fusion, dictionary learning, sparse representation, multi-scale spatial frequency

## Abstract

Training a good dictionary is the key to a successful image fusion method of sparse representation based models. In this paper, we propose a novel dictionary learning scheme for medical image fusion. First, we reinforce the weak information of images by extracting and adding their multi-layer details to generate the informative patches. Meanwhile, we introduce a simple and effective multi-scale sampling to implement a multi-scale representation of patches while reducing the computational cost. Second, we design a neighborhood energy metric and a multi-scale spatial frequency metric for clustering the image patches with a similar brightness and detail information into each respective patch group. Then, we train the energy sub-dictionary and detail sub-dictionary, respectively by K-SVD. Finally, we combine the sub-dictionaries to construct a final, complete, compact and informative dictionary. As a main contribution, the proposed online dictionary learning can not only obtain an informative as well as compact dictionary, but can also address the defects, such as superfluous patch issues and low computation efficiency, in traditional dictionary learning algorithms. The experimental results show that our algorithm is superior to some state-of-the-art dictionary learning based techniques in both subjective visual effects and objective evaluation criteria.

## 1. Introduction

In recent decades, various medical imaging techniques have emerged and numerous medical images have been provided. Different modal medical images can only offer a limited characteristic description about human organs due to the diverse imaging mechanism [1]. Medical image fusion technology can integrate the complementary information of multiple single-modal medical images acquired by different imaging sensor modalities and provide a more precise, reliable, and better description of lesions [2]. This technology has witnessed various applications in real products such as medical assistance, as it can provide doctors a comprehensive description on disease tissues, so as to develop appropriate treatment plans and improve diagnostic accuracy.

In past decades, various research efforts have been made to develop an effective medical image fusion technique [3]. In general, according to the characteristics of the medical images, multi-scale transform (MST) based image fusion methods have been the most popular trend due to their simplicity, feasibility, and effectiveness in implementation [4,5,6]. A number of typical MSTs were developed, for example: Stationary Wavelet Transform (SWT) [7], Dual-Tree Complex Wavelet Transform (DTCWT) [8], Curvelet Transform (CVT) [9], Contourlet Transform (CT) [10], Nonsubsampled Contourlet Transform (NSCT) [11,12], etc. The MST-based methods first decompose the source image into a series of sub-images to express the base and detail information with different levels and directions and then fuse the lowpass and high-pass coefficients according to the designed fusion rules respectively. Finally, the fusion result can be obtained by performing the inverse MST, although the MST-based methods can effectively extract some important features from the source image into the fused image. However, these methods cannot adaptively express the content of the image, which heavily limits their fusion performance, meanwhile, each MST tool has its own merits and limitations depending on the context of input images [3]. Furthermore, some useful information may be inevitably lost in the process of decomposition and reconstruction, and the suboptimal results would be generated. Thus choosing an optimal MST is not an obvious and trivial task as it relies on scene contexts and applications.

In order to address the deficiencies of MSTs based methods and obtain an encouraging fusion performance, a plethora of carefully designed medical image fusion algorithms have been proposed, such as neural network (NN) based methods [13,14,15], principal component analysis (PCA) based techniques [16], and mathematical morphology (MM) based methods [17] and so on. However, the NN based method relies too much on a large number of parameters manually set, which is not conducive to the adaptive implementation of the fusion process. The PCA based technique is prone to produce the drawback of spectral distortion. Although the MM based algorithm plays an important role in image fusion, some of the details in source image may be smoothed in the final fusion result, which would affect the fusion performance and hinder the applications of them.

Sparse representation (SR) theory can describe and reconstruct images in a sparse and efficient way by linear combination of sparse coefficients and overcomplete dictionary [18]. In recent years, SR theory has been rapidly developed and successfully applied in many image processing applications, such as image super-resolution reconstruction [19,20], image feature extraction [21], image denoising [22,23], pedestrian re-identification [24,25], image classification [26], and many other fields. At the same time, it has been successfully applied to many image fusion fields and achieved some satisfactory results [27,28,29]. In SR, the overcomplete dictionary is of important significance in medical image representation. It also plays a key role in affecting the quality of fused image. Yang and Li [30] took the first step for utilizing SR theory into the field of multi-focus image fusion, in which DCT was used as a fixed dictionary. Subsequently, they proposed a SR based on Simultaneous Orthogonal Matching Pursuit (SOMP) and successfully applied for multimodal image fusion [31]. Jiang et al. [17] regarded the image as a superposition of two different components of cartoon and texture, respectively, they employed Curvelet and DCT basis as two different dictionaries to express their respective information, and proposed an image fusion method based on SR and morphological component analysis. However, owing to the fact that dictionaries produced by these methods are constructed by the fixed base functions with poor adaptability, this is not an effective way to describe the complex structure information of medical images [18].

Relative to analytical fixed dictionaries, dictionary learning based methods use a small amount of atoms from a trained dictionary instead of a predefined one [27], which can produce state-of-the-art results in many image processing and recognition tasks [2]. Learning from the training sample images to obtain an informative and compact overcomplete dictionary can enhance the adaptability of the dictionary, as well as accurately express the medical image information. In order to produce better fusion quality, many recent dictionary learning based fusion algorithms have been proposed [15]. Meanwhile, some SR based methods that directly train the source images to train the overcomplete dictionary can generate promising fusion results [31]. However, many methods suffered from a general problem that taking all of the patches for dictionary learning, which will unavoidable, would introduce lots of unvalued and redundant information during dictionary training and decrease the fusion performance in medical image fusion. To address this shortcoming in the literature [32], Zhu et al. developed a local density peak-clustering algorithm to refine the process of patch selection, and then established a compact dictionary by K-SVD algorithm. Kim et al. [33] employed a joint clustering technique to classify image patches according to their similar structures, the overcomplete dictionary can be obtained by combing principal components of them. In order to capture the intrinsic features of the image and preserve the hierarchical structure of stationary wavelets, Yin [34] designed a joint dictionary learning method based on all base subbands of stationary wavelets. Liu et al. [35] proposed an adaptive SR method for simultaneous fusion and denoising the images. In this method, they classified a lot of high-quality image patches with gradient information into several categories, and then trained each sub-dictionary of them. Qi et al. [36] proposed an entropy based image fusion method. In this method, the source images were decomposed into low frequency and high frequency images respectively, then the weighted average scheme was utilized to fuse the low-frequency part and the entropy based dictionary learning technique was introduced to fuse the high-frequency part. However, the average scheme for the low-frequency component would inevitably lose some energy of the input image, which would decrease some useful brightness information in fused results. Although comparing with some traditional medical image fusion techniques, the medical image feature can be represented effectively and completely in these methods [32,33], the fusion performance remaining has much room for improvement.

The purpose of the medical image fusion technique is to retain useful information from source images into a fused result as much as possible [37]. To address the shortcomings of low-learning efficiency and weak dictionary expression ability in traditional dictionary learning based algorithms, this paper proposes a novel dictionary learning method based on the brightness and detail clustering for medical image fusion. Our method consists of three steps, the enhancement and multi-scale sampling on images, patch feature classification and dictionary construction, and image fusion. Firstly, we enhance the details of the source images by multi-layer filtering technology, which can significantly improve the information expression ability of dictionary. In the meantime, we conduct a multi-sampling scheme on enhanced images. This operation can significantly increase the richness of patches while decreasing computation load in the dictionary learning stage. Secondly, in order to obtain a fused result with abundant brightness and detail features, i.e., the successful expression of the underlying visual features of the medical image, we developed two feature clustering criteria to classify all patches into two categories including a brightness group and a details group, and construct the energy sub-dictionary and the detail sub-dictionary by K-SVD algorithm. These sub-dictionaries can be directly built by the final compact and informative dictionary. Finally, the sparse coefficient can be calculated by the orthogonal matching pursuit (OMP) algorithm [38], and then reconstructed to the fused images. The main contributions of this paper can be elaborated as follows:

1. We conduct multi-level neighbor distance filtering to enhance the information and take multi-scale sampling to realize the multi-scale expression of images, which can make image patches more informative and flexible, while not increasing the computational complexity in the training stage.
2. Based on the characteristics of the human visual system processing medical images, we develop novel neighborhood energy and a multi-scale spatial frequency to cluster brightness and detail patches, and then train the brightness sub-dictionary and detail sub-dictionary, respectively.
3. A feature discriminative dictionary is constructed by combing the two sub-dictionaries. The final dictionary contains important brightness and detail information, which can effectively describe the useful feature of medical images.

The rest of the paper is organized as follows: The proposed dictionary learning method for medical image fusion is described in detail in Section 2, including reviews the basic theory of SR, dictionary learning and SR subsections. The experiments and results analysis are presented in Section 3. Conclusions and discussion are summarized in Section 4.

## 2. Proposed Framework 

In this section, we present the proposed technique in detail. There are three sub-sections in this part, which include the theory of SR, dictionary learning and image fusion. In the dictionary step, we propose two techniques for images to generate informative training images. Then, the image patches are clustered into brightness and detail groups based on the neighborhood energy and the multi-scale spatial frequency, respectively. The K-SVD algorithm is employed to construct a brightness-based sub-dictionary and a detail-based sub-dictionary, and the final dictionary comsists of the sub-dictionaries. In the image fusion step, a SR model is well established and the sparse coefficients can be calculated by OMP, then generate the final fused image. 

### 2.1. Sparse Representation 

For a signal $y=(y_{1},y_{2},\ldots y_{n}),$ $y\in {\mathbb{R}}^{n}$, the basic assumption in SR theory is that y can be approximately represented as a linear combination of a set of base signals ${\left\{d_{i}\right\}}_{i=1}^{m}$ from a redundant dictionary $D\in {\mathbb{R}}^{n\times m}$ (*n* < *m*), the signal y can be expressed as

(1) $$y=\sum_{i=1}^{m}d_{i}\alpha_{i}=D\alpha$$

where $\alpha =(\alpha_{1},\alpha_{2},\ldots \alpha_{n})$ is the unknown sparse coefficient vector, *d_i_* is an atom of D. Given a signal y and an overcomplete dictionary D, the process of finding the sparse coefficient α is called sparse representation. The goal of SR is to calculate the sparsest α, which contains the fewest non-zero entries. However, this is an underdetermined problem due to the existence of overcomplete D. Generally, the sparsest α can be obtained by solving the following sparse model:

(2) $$\hat{\alpha }=\underset{\alpha }{\arg\min}{\left\|\alpha \right\|}_{0},\;s.t.{\left\|y-D\alpha \right\|}_{2}^{2}<\varepsilon$$

where ${\left\|•\right\|}_{0}$ denotes the $l_{0}$ norm that counts the number of non-zero entries, *ε* > 0 is an error tolerance. Equation (2) is a non-deterministic polynomial-hard (NP-hard) problem which can be solved by the greedy approximation approach OMP [38].

### 2.2. Proposed Dictionary Learning Approach 

**Figure 1:**
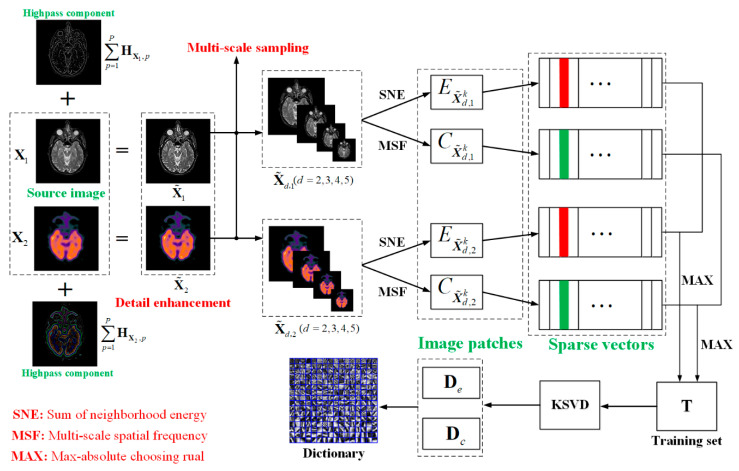
Procedure of dictionary learning.

For SR based medical image fusion, the ability of information expression of dictionary has a direct, great impact on the fusion results. Numerous studies [32,33,35] indicate that the dictionary obtained by the traditional training methods cannot generate impressive fusion results because of the weak expression ability. In order to construct an informative as well as compact dictionary, we propose a new dictionary learning method illustrated in Figure 1. In the first stage, two effective techniques including detail enhancement and multi-scale sampling are developed for improving the qualities of image patches. In the second stage, a neighborhood energy criterion is introduced to cluster the brightness patches and a multi-scale spatial frequency criterion is proposed to cluster the edge detail patches, and then generate two high-quality training sets. In the last stage, the brightness sub-dictionary and detail sub-dictionary can be constructed by training the two categories of patches using K-SDV, then the overcomplete dictionary is obtained by the combination of the sub-dictionaries. The detailed analyses of these three sub-sections are shown in the rest of the sub-sections.

#### 2.2.1. Detail Enhancement

**Figure 2:**
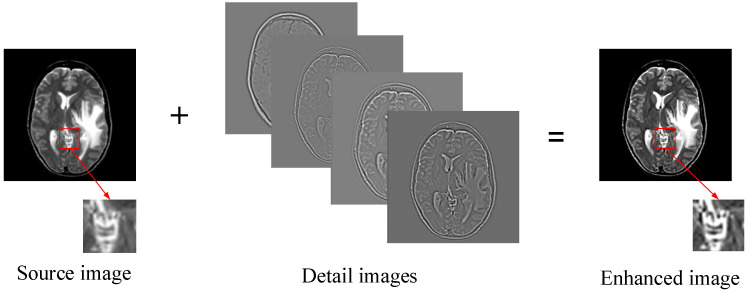
Enhancement of training samples.

We employ the way of online learning to obtain the overcomplete dictionary. The advantage is that it can directly utilize useful information from source images and can be beneficial to enhance the expressive ability of the dictionary. However, the general way of dividing the image into a series of overlapping patches is directly performed on the pre-training images, which would lead to some un-conspicuous problems. When the details of certain regions in the source image are relatively weak, some important information in this region could not be well expressed in dictionary training. To address this problem, we develop a detail enhancement technique shown in Figure 2. The high-pass detail information with different levels are first extracted by simple multiple filtering, and then superimposes into the source images, which can be mathematically expressed as:

(3) $${\tilde{X}}_{l}(i,j)=X_{l}(i,j)+\sum_{p=1}^{P}H_{X_{l},p}(i,j)$$

(4) $$H_{X_{l},p}=ND*X_{l}$$

where $X_{l}=X_{1},\;X_{2},\ldots$ denote the *l*-th source image, if only two source images in medical image fusion procedure, l=1,2, ${\tilde{X}}_{l}$ is the detail enhanced version of $X_{l}$, $H_{X_{l,p}}$ is the *p*-th level highpass detail information image of $X_{l}$. 

*ND* denotes the Neighbor Distance filter, some studies [39,40] demonstrated that *ND* can effectively extract and express the high frequency information of image, so we use *ND* to extract details in this step, more detailed introduction about *ND* can be found in [40]. In addition, it should be noted that this paper enhances the source image when training them to generate a strong overcomplete dictionary, rather than enhanced the source images during the fusion process.

As can be seen from Figure 2, by performing the process of detail enhancement, the weak information such as boundary, brightness and texture et al. of the source images are significantly enhanced. For the convenience of comparison, a same region from source image and enhanced image is selected by the red rectangle and enlarged and placed in the lower right of them. The enhanced image can highlight the unobvious but significant information, and express them into patches, which could be beneficial to constructing a strong training set.

#### 2.2.2. Multi-Scale Sampling (MSS)

**Figure 3:**
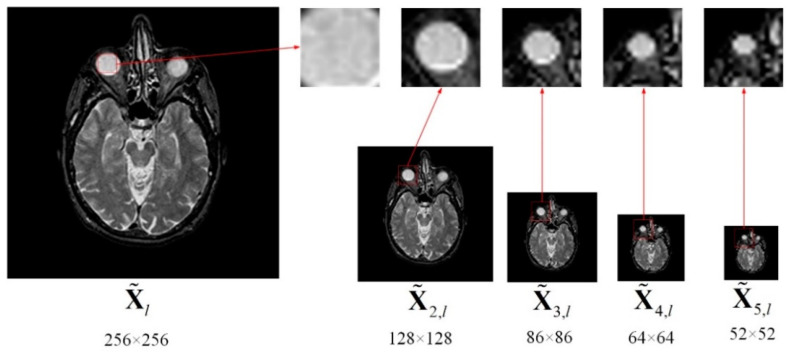
Sampling.

For dictionary learning, the traditional way of constructing a training set is first dividing the images into a series of overlap patches by a fixed size, and then pull them into a vector respectively. However, the fixed size of image patch usually cannot comprehensive describe image information, especially small target information in medical images. Generally, a large size of image patch is more likely to improve robustness and it can illustrate the shape and location of object, but the description about the details are insufficient, and vice versa. Therefore, choosing a suitable patch size is significant for improving the informative ability of the training set. To integrate the advantages of each patch with different sizes, we present a Multi-scale Sampling (MSS) scheme to obtain a stronger training set. MSS can not only improve multi-scale properties of the training set but also reduce the dictionary training time. The MSS can be obtained by the follows

(5) $${\tilde{X}}_{d,l}=\mathrm{downsample}({\tilde{X}}_{l},d)$$

where ${\tilde{X}}_{d,l}$ is the sampling version of ${\tilde{X}}_{l}$ by downsampling rate of d (d=1,2,…), obviously, ${\tilde{X}}_{l}={\tilde{X}}_{1,l}$. As can be seen from Figure 3. A series of different images with various smaller size of source can be generated. 

To facilitate the explanation, the white circular areas in each of ${\tilde{X}}_{d,l}$ images in Figure 3 are marked by same-sized red rectangles. We can notice that the contour and details for the same region in diverse images with different size are significantly different. By performing MSS, the useful information can be comprehensively utilized from coarse to fine with a different scale, then the informative image patches can be obtained by dividing.

More importantly, MSS can increase the computation efficiency due to the downsampling operation. For example, as illustrated in Figure 3, the size of the input image ${\tilde{X}}_{l}$ is 256 × 256, when the downsampling rate *d* is set to 2, 3, 4 and 5, respectively, we can generate ${\tilde{X}}_{2,l}$, ${\tilde{X}}_{3,l}$, ${\tilde{X}}_{4,l}$ and ${\tilde{X}}_{5,l}$, the size of each of them is smaller than ${\tilde{X}}_{l}$. Using several images processed by MSS with different sampling rates instead of source images to construct a training set would not decrease dictionary quality, while reducing dictionary learning time. In order to analyze quantitatively, as shown in Table 1, we calculate the number of patches from the upper left to the lower right, which is produced by the patch size 8 × 8 and a step length of two (pixels overlapping is six). As can be seen from Table 1, the summation of patches obtained by the four images $\left\{{\tilde{X}}_{d,l}\right\}$ (d=2,3,4, 5) is much smaller than ${\tilde{X}}_{l}$, which means the process of MSS can significantly reduce the training data, and then decrease the computation complexity in dictionary learning.

#### 2.2.3. Sum of Neighborhood Energy (SNE)

**Figure 4:**
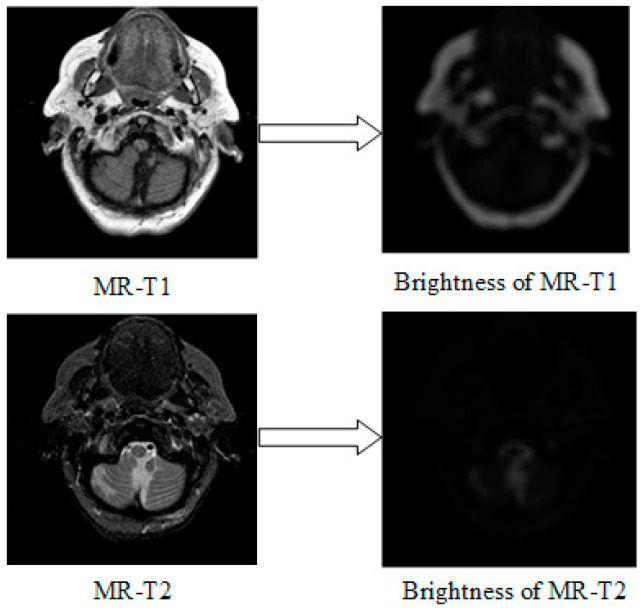
Images (**left**) and their brightness (**right**) by SNE.

Brightness information and detailed information are two important manifestations for medical image features. Thus, detecting the brightness and detail feature from the source images into the fused image is necessary to produce encouraging fusion results. Considering the energy correlation between pixels in medical images, the sum of neighborhood energy (SNE) is developed to evaluate the brightness of the central pixel, as follows:

(6) $$e(i,j)=\sum_{m=-(M-1)/2}^{(M-1)/2}\sum_{n=-(N-1)/2}^{(N-1)/2}{\tilde{X}}_{d,l}{\left(j+m,j+n\right)}^{2}$$

where e(i,j) denote the SNE of ${\tilde{X}}_{d,l}$ centered at pixel (i,j); ${\tilde{X}}_{d,l}(i+m,j+n)$ is the pixel value of ${\tilde{X}}_{d,l}$ at pixel (i+m,j+n); M×N indicate the size of neighborhood. The MR-T1/MR-T2 images and their corresponding brightness images are displayed in Figure 4.

#### 2.2.4. Multi-Scale Spatial Frequency (MSF)

In medical images, the spatial frequency (SF) of the centered pixel can reflect the difference between the pixel and the surrounding pixel. Thus, SF of an image can be used to express the richness degree of the image detail information. Considering the correlation between pixels, a neighborhood based SF is usually employed, which is defined as:

(7) $$SP_{{\tilde{X}}_{d,l}}(i,j)=\sum_{m=-(M-1)/2}^{m=(M-1)/2}\sum_{n=-(N-1)/2}^{n=-(N-1)/2}\left[{\tilde{X}}_{d,l}(i+m,j+n)-X(i,j)\right]$$

where $SP_{{\tilde{X}}_{d,l}}(i,j)$ is the value of SF of ${\tilde{X}}_{d,l}$ centered at (i,j), M×N is the size of neighborhood.

In practical applications, to reduce the computational complexity, the size of the neighborhood is usually set to M = N. However, this technique in Equation (7) is sensitive to the neighborhood size, especially for the dark and low-contrast regions. To address this problem, we propose a novel detail detection technique called Multi-scale spatial frequency (MSF), The MSF of an image is mathematically defined as

(8) $$\begin{array}{r} MSF_{{\tilde{X}}_{d,l}}(i,j)=\varepsilon_{1}\left|SP_{{\tilde{X}}_{d,l}}^{r_{1}}(i,j)-SP_{{\tilde{X}}_{d,l}}^{r_{2}}(i,j)\right|\\ +\varepsilon_{2}\left|SP_{{\tilde{X}}_{d,l}}^{r_{1}}(i,j)-SP_{{\tilde{X}}_{d,l}}^{r_{3}}(i,j)\right| \end{array}$$

here

(9) $$SP_{{\tilde{X}}_{d,l}}^{r}(i,j)=\sum_{m=-(r-1)/2}^{m=(r-1)/2}\sum_{n=-(r-1)/2}^{n=-(r-1)/2}\left[{\tilde{X}}_{d,l}(i+m,j+n)-{\tilde{X}}_{d,l}(i,j)\right]$$

where, r ($r=r_{1},r_{2},r_{3}$) is the scale factor, r×r denotes the size of neighborhood,$\varepsilon_{1}$ and $\varepsilon_{2}$ are weighting parameter given by the user. $SP_{{\tilde{X}}_{d,l}}^{r}(i,j)$ is the *r*-scale SF of ${\tilde{X}}_{d,l}$ at pixel (i,j), $MSF_{{\tilde{X}}_{d,l}}(i,j)$ is the MSF of ${\tilde{X}}_{d,l}$ at pixel (i,j).

#### 2.2.5. Clustering and Dictionary Learning

We classify all patches into two groups by taking SNE and MSF as the feature metrics for clustering. The patch clustering and dictionary learning consist of the following five steps.

• *Step 1*: Patches Collection

Adopt the slide window technique to divide each of ${\tilde{X}}_{d,l}$ into a suite of image patches with size ρ×ρ from upper left to lower right, and then overlapping each of them, the step length between two adjacent patches is δ pixels. The patches extraction results are as follows:

(10) $${\tilde{X}}_{d,l}={\tilde{X}}_{d,l}^{1}\cup {\tilde{X}}_{d,l}^{2}\cup \ldots \cup {\tilde{X}}_{d,l}^{k}\cup \ldots \cup {\tilde{X}}_{d,l}^{K}$$

where ∪ denotes merging each of them, ${\tilde{X}}_{d,l}^{k}$ is the *k*-th patch of ${\tilde{X}}_{d,l}$, *K* is the total number of image patches.

• *Step 2*: Calculate patch feature by SNE and MSF

Calculate SNE and MSF of each image patch respectively, a series of brightness dominant patches and a number of detail dominant patches are obtained. According to Equation (6), the energy feature of patch ${\tilde{X}}_{d,l}^{k}$ can be calculated as following:

(11) $$E_{{\tilde{X}}_{d,l}^{k}}=\sum_{(x,y)\in {\tilde{X}}_{d,l}^{k}}e_{{\tilde{X}}_{d,l}^{k}}(x,y)$$

where $e_{{\tilde{X}}_{d,l}^{k}}(x,y)$ is the brightness level of the *k*-th patch in ${\tilde{X}}_{d,l}$ at pixel (x,y), $E_{{\tilde{X}}_{d,l}^{k}}$ denotes the sum of brightness information of patch ${\tilde{X}}_{d,l}^{k}$. Similarly, the sum of detail information contained in patch ${\tilde{X}}_{d,l}^{k}$ can be generated by Equation (8):

(12) $$C_{{\tilde{X}}_{d,l}^{k}}=\sum_{(x,y)\in {\tilde{X}}_{d,l}^{k}}c_{{\tilde{X}}_{d,l}^{k}}(x,y)$$

where $c_{{\tilde{X}}_{d,l}^{k}}(x,y)$ denotes the detail level of the *k*-th patch in ${\tilde{X}}_{d,l}$ at pixel (x,y), $C_{{\tilde{X}}_{d,l}^{k}}$ indicates the sum of the detail information of patch ${\tilde{X}}_{d,l}^{k}$

• *Step 3*: Construct training sets

Rearrange each patch ${\tilde{X}}_{d,l}^{k}$ into a column vector ${\bar{X}}_{d,l}^{k}$, and then construct a brightness training set by selecting the corresponding image patches containing the best brightness activity level by the following rules:

(13) $$T_{e}=\{{\bar{T}}_{1},{\bar{T}}_{2},\ldots {\bar{T}}_{k},\ldots ,{\bar{T}}_{K}\}$$

(14) $${\bar{T}}_{k}=\max\{E_{{\tilde{X}}_{d,1}^{k}},E_{{\tilde{X}}_{d,2}^{k}},\ldots E_{{\tilde{X}}_{d,l}^{k}},\ldots \}$$

where $T_{e}$ is the brightness dominant training set, which composes the image patches with a better brightness feature, and ${\bar{T}}_{k}$ denotes that the *k*-th patch from $\left\{E_{{\tilde{X}}_{d,1}^{k}},E_{{\tilde{X}}_{d,2}^{k}},\ldots E_{{\tilde{X}}_{d,l}^{k}},\ldots \right\}$ has the largest SNE value among them. Similarly, construct a detail training set by choosing the corresponding image patches containing the richest detail activity level by the following rules:

(15) $$W_{c}=\{{\bar{W}}_{1},{\bar{W}}_{1},\ldots {\bar{W}}_{k},\ldots ,{\bar{W}}_{K}\}$$

(16) $${\bar{W}}_{k}=\max\{C_{{\tilde{X}}_{d,1}^{k}},C_{{\tilde{X}}_{d,2}^{k}},\ldots C_{{\tilde{X}}_{d,l}^{k}},\ldots \}$$

where $W_{c}$ is the detail dominant training set which composes of image patches with better detail feature, and ${\bar{W}}_{k}$ means that the *k*-th patch from $\left\{C_{{\tilde{X}}_{d,1}^{k}},C_{{\tilde{X}}_{d,2}^{k}},\ldots C_{{\tilde{X}}_{d,l}^{k}},\ldots \right\}$ has the largest MSF value is selected. 

• *Step 4*: Sub-dictionaries learning

The brightness sub-dictionary $D_{e}$ can be generated by calculating the objective function in Equation (15) by using K-SVD [41],

(17) $$\{D_{e},\alpha_{e}\}\underset{D_{e},\alpha_{e}}{=\arg\min}\left\{{\left\|T_{e}-D_{e}\alpha_{e}\right\|}_{F}^{2}+{\left\|\alpha_{e}\right\|}_{1}\right\}$$

where $D_{e}$ and $\alpha_{e}$ are the brightness sub-dictionary and its sparse coefficient, respectively. 

To avoid this, a same patch is used in the brightness set as well as the detail set. At the same time, we add the following constraint

(18) $$E_{{\tilde{X}}_{d,l}^{k}}-C_{{\tilde{X}}_{d,l}^{k}}\neq 0$$

The detail sub-dictionary $D_{c}$ is obtained by performing K-SVD on detail training set $W_{c}$ as follows

(19) $$\{D_{c},\alpha_{c}\}\underset{D_{c},\alpha_{c}}{=\arg\min}\left\{{\left\|W_{c}-D_{c}\alpha_{c}\right\|}_{F}^{2}+{\left\|\alpha_{c}\right\|}_{1}\right\}$$

where $D_{c}$ and $\alpha_{c}$ are the detail sub-dictionary and its sparse coefficient, respectively.

• *Step 5*: Construct the final dictionary

The final informative, compact and discriminative overcomplete dictionary is obtained by the combination of the sub-dictionaries $D_{e}$ and $D_{c}$ in Equation (20)

(20) $$D=(D_{e},D_{c})$$

The schematic diagram of the proposed dictionary learning is illustrated in Figure 1. Moreover, the formalized mechanism of the dictionary learning algorithm is described in Algorithms 1. For simplicity, we take two source images as an example to discuss, and it can easily be extended to the case of more than two source images.

**Algorithm 1** The proposed dictionary learning algorithm**Inputs**: Two group of images $\left\{{\tilde{X}}_{d,l}\right\}$ (l=1,2)(1) Extract patches of $\left\{{\tilde{X}}_{d,1}\right\}$ and $\left\{{\tilde{X}}_{d,2}\right\}$ from upper left to lower right.(2) Patch classification based on SNE and MSF in Equations (6) and (8), respectively.(3) Construct two training sets $T_{e}$ and $W_{c}$ by Equations (13) and (15), respectively.(4) Obtain the two sub-dictionaries $D_{e}$ and $D_{c}$ by solve Equations (17) and (19), respectively.(5) Generate the final dictionary D by Equation (20)**Output**: The overcomplete dictionary D

### 2.3. Image Fusion

**Figure 5:**
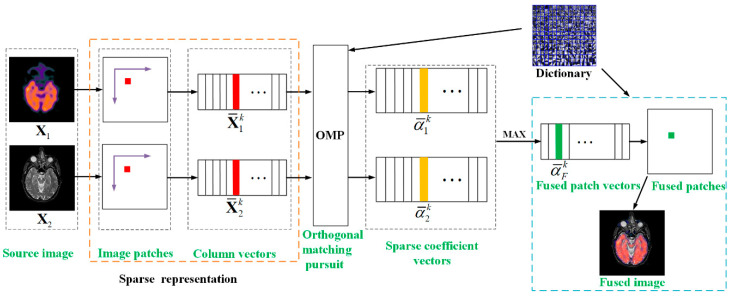
Framework of the proposed image fusion.

The whole image fusion schematic diagram is shown in Figure 5. In the sparse coding process, each source image $X_{l}$ is first divided into a series of image patches of size ρ×ρ from upper left to lower right with an overlapping step length of δ pixels, and then rearranging them into column vectors ${\bar{X}}_{l}^{k}$ (k=1,2,…,K, l=1,2,…,n), where K and n are the number of patches and source images, respectively. ${\bar{X}}_{l}^{k}$ denotes the *k*-th patch’ column vector of image $X_{l}$. Calculate the sparse coefficient vectors $\left\{{\bar{\alpha }}_{1}^{k},{\bar{\alpha }}_{2}^{k},\ldots ,{\bar{\alpha }}_{n}^{k}\right\}$ of source images by using OMP algorithm and the following underdetermined equation can be solved

(21) $$\hat{\alpha }=\underset{\alpha }{\arg\min}\left\{{\left\|y-D\alpha \right\|}_{2}^{2}+{\left\|\alpha \right\|}_{1}\right\}$$

where $\hat{\alpha }=\{{\bar{\alpha }}_{1}^{k},{\bar{\alpha }}_{2}^{k},\ldots ,{\bar{\alpha }}_{n}^{k}\}$, **y** is the sparse coefficient vectors $\left\{{\bar{X}}_{1}^{k},{\bar{X}}_{2}^{k},\ldots ,{\bar{X}}_{n}^{k}\right\}$. By employing the ‘‘max-absolute choosing’’ rule to obtain the fused sparse vector ${\bar{\alpha }}_{F}^{k}$

(22) $${\bar{\alpha }}_{F}^{k}=\max\{{\bar{\alpha }}_{1}^{k},{\bar{\alpha }}_{2}^{k},\ldots ,{\bar{\alpha }}_{n}^{k}\}$$

The *k*-th patch’ column vector of fused image can be generated by combing D and ${\bar{\alpha }}_{F}^{k}$

(23) $${\bar{F}}_{k}=D{\bar{\alpha }}_{F}^{k}$$

Reshape all of the column vectors ${\bar{F}}_{k}$ into image patches $F_{k}$ and then plug each of $F_{k}$ into its corresponding original position, the final fused image F can be obtained:

(24) $$F=\overset{K}{\underset{k=1}{\cup }}F_{k}$$

## 3. Experiments and Analysis

In this section, we present four subsections to analyze and verify the superiority of the proposed medical image fusion scheme. We first illustrate the detail experimental setups including test methods and parameters setting in Section 3.1. Then, the test images in experiments are displayed and briefly introduced in Section 3.2. We evaluate the fusion results of different methods based on their visual performance and quantitative assessment in Section 3.3 and Section 3.4. The computation efficiency analysis is discussed in Section 3.5. Finally, we extend our method into other type of image fusion applications in Section 3.6.

### 3.1. Test methods and Parameters Setting

In order to verify the effectiveness of the proposed method, six representative medical image fusion algorithms are selected to compare with the proposed algorithm. The first three compared methods are MST based: DTCWT, CVT and NSCT. The others are three state-of-the-art medical image fusion methods including the adaptive SR (Liu-ASR) proposed by Liu et al. [35], the Kim [33] method, which is based on clustering and principal component analysis methods, and the K-SVD based fusion technique developed by Zhu et al. [32]. The latter three carefully design algorithms and mainly paid their attention to the novel ways for training an informative overcomplete dictionary, they can produce the representative fusion performance for the field of medical image fusion in a few years.

For the parameters setting, the first layer of DTCWT uses ‘LeGall 5-3’ filter, and the other layer employs ‘Qshift-06’ filter. The NSCT method employs the ‘pyrexc’ filter as a pyramid filter, and the ‘vk’ filter as the directional filter, which is decomposed into four layers, and the number of directions from coarse to fine is 4, 8, 8, and 16, respectively. These setting can generate the best fusion performance [3]. For all the MST based methods, they fuse high-frequency coefficients and low-frequency coefficients by “max-absolute” and “weighted averaging” scheme. Furthermore, a 3×3 consistency verification check [42] is adopted to fuse the high-frequency coefficients. The default parameters given by the respective authors are used for the Liu-ASR, Zhu and Kim methods.

For the proposed method, during the training dictionary stage, the size of patch is conventionally set to 8×8, and the overlapping pixels is 6. For the detail enhancement, the number of level P is set to 4 in Equation (3). For the MSS, the downsampling rate is set 3, 4, 5 and 6, respectively in Equation (5). As a consequence, the proposed dictionary learning based method can generate high fusion performance while keep lower computation efficiency. Meanwhile, due to the strong flexibility of multiscale property in MSS, the MSS scheme can also well address the case of small size source images. The size of the neighborhood of the SNE in Equation (6) is set to 11 × 11. For MSF, the three scale factors $r_{1}$, $r_{2}$ and $r_{3}$ are set to 7, 11 and 15, and the weight coefficients $\varepsilon_{1}$ and $\varepsilon_{2}$ are set to 0.33 and 0.67 in Equation (8), respectively. In the sparse representation stage, the reconstruction error is 0.1, and the number of iterations is 50. The setting of the parameters of the proposed algorithm can generate the best fusion results for the experimental images in this paper.

### 3.2. Test Images

**Figure 6:**
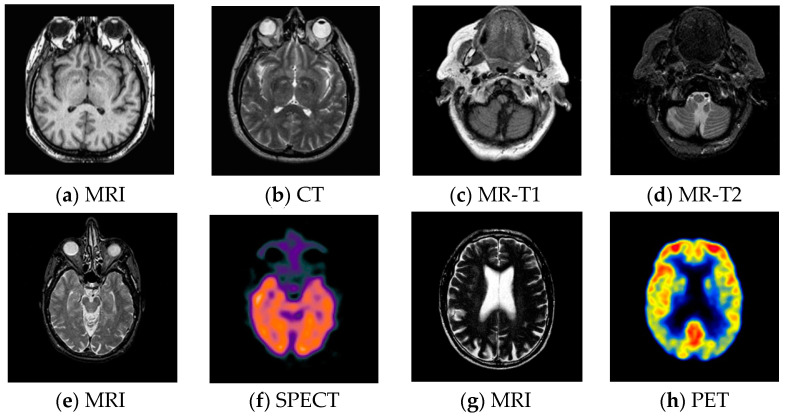
Pairs of medical source images: (**a**,**b**) MRI/CT images, (**c**,**d**) MR-T1/MR-T2 images; (**e**,**f**) MRI/SPECT images; (**g**,**h**) MRI/PET images.

For pixel level multi-modality medical image fusion, most of the test images generally can be obtained at http://www.imagefusion.org and http://www.med.harvard.edu/aanlib/home.html. To demonstrate the effectiveness of the proposed method, we utilize four pairs of real multimodal medical images shown in Figure 6 to evaluate the algorithm in our experiments, the size of all of them is 256 × 256, and all of them are perfectly registered, which means that the objects in each set of images are geometrically aligned. These medical images include MRI, CT, MR-T1, MR-T2, SPECT and PET, the characteristics of each of them are summarized as following.

1. CT has a shorter imaging time and a higher spatial resolution, whereas it provides soft tissue information with low contrast.
2. MRI can clearly display the soft tissue information of the human body, but it is hard to reflect the dynamic information of the metabolic activity in human body.
3. MR-T1 image is sensitive to observe the anatomy, while the MR-T2 image can detect the tissue lesions.
4. SPECT can show the biological activities of cells and molecules, but it is difficult to distinguish human organ tissues due to the low image quality of SPECT.
5. PET can reflect the metabolic activity information of human tissues and organs at the molecular level, but the spatial resolution of PET is relatively low.

In our experiments, all of the tests are implemented in Matlab 2016a using a desktop with a Core (TM) i7-3770 CPU, 3.40GHz, 8 CPUs and 12G RAM.

### 3.3. Fusion Results by Subjective Visual Effects Analysis

**Figure 7:**
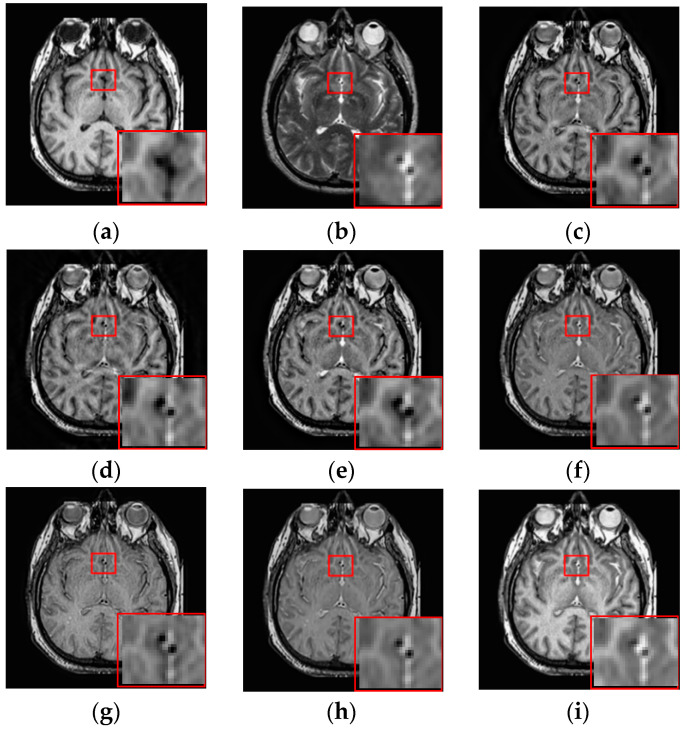
Fused results of MRI/CT medical images by different methods. (**a**) MRI; (**b**) CT; (**c**) DCTWT; (**d**) Curvelet; (**e**) NSCT; (**f**) Liu-ASR; (**g**) Kim; (**h**) Zhu; (**i**) Proposed.

The results of the first set of experiments are shown in Figure 7, and the source MRI and CT images are listed in Figure 7a,b. The fused images obtained by the DCTWT, Curvelet, NSCT, Liu-ASR, Kim, Zhu, and the proposed method are displayed in Figure 7c–i, respectively. To facilitate subjective visual comparisons, local regions enclosed by red colored rectangular boxes in Figure 7 are enlarged and presented in the bottom right corners of their respective images. We can notice that the diverse fusion performance generated by different methods in retaining the brightness information and edge detail the information of source medical images. Despite the MST methods expressing source information with different scales and directions, all of the results obtained by DTCWT, Curvelet and NSCT in Figure 7c–e still produce some distortion, which heavily decreases the qualities of the medical images. Meanwhile, after careful observation, we can find the fusion results generated by Liu, Kim and Zhu can preserve details well from MRI. However, the brightness active level is relatively lower than the source CT image, which indicates the loss of useful information. It can be seen from Figure 7i that the fusion result obtained by the proposed method has the best performance in terms of retaining brightness information and detail information, which indicates that the visual effect of the method is the best.

**Figure 8:**
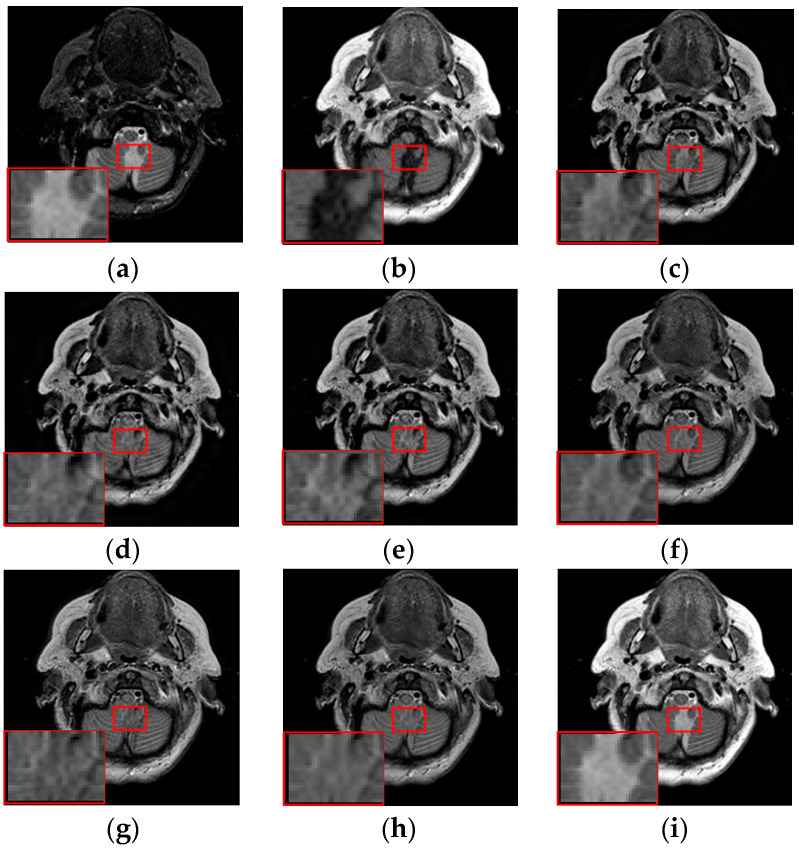
Fused results of MR-T1/MR-T2 medical images by different methods. (**a**) MR-T1; (**b**) MR-T2; (**c**) DCTWT; (**d**) Curvelet; (**e**) NSCT; (**f**) Liu-ASR; (**g**) Kim; (**h**) Zhu; (**i**) Proposed.

The fusion results of different methods about “MR-T1/MR-T2” (see Figure 8a,b) are shown in Figure 8c–i. For this experiment, the detail information (shape, edge, texture et. al) in the red rectangle is mainly from the MR-T1 image while the energy information (brightness, contrast et.al) mainly from MR-T2 image. Among these fusion results, it can be seen that the details of the fusion results produced by the DTCWT, Curvelet and NSCT methods are severely damaged, especially the Curvelet method. Although the Liu-ASR, Kim and Zhu methods can relatively effectively protect the edge details of the source image, they do not protect the contrast of the image well. This is very disadvantageous for medical images with high-quality requirements, and is not conducive to subsequent medical image processing and recognition tasks. 

By comparison, our fusion result (Figure 8i) can not only effectively protect the edge detail information of the source image, but also maintain the contrast of the source image, which is mainly due to the detail enhancement processing of training set and the clustering techniques to classify brightness and detail groups before dictionary learning. At the same time, little artificial false information is introduced in our fusion result, which means that the visual effect of the proposed method outperforms others methods in this experiment.

**Figure 9:**
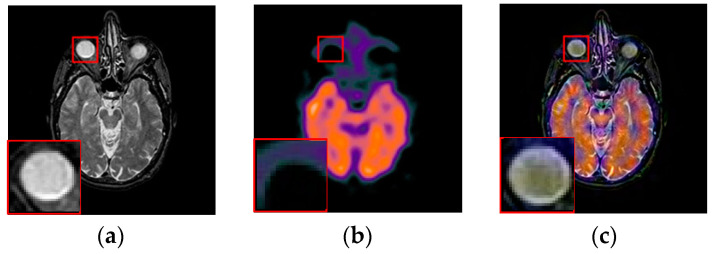

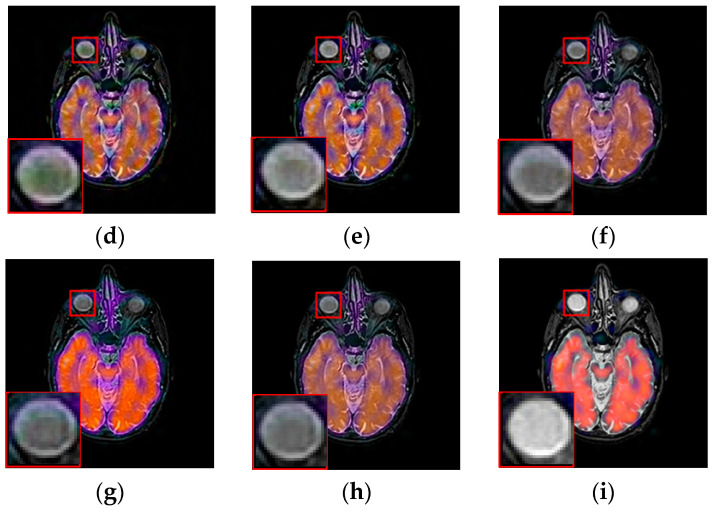
Fused results of MRI/SPECT medical images by different methods. (**a**) MRI; (**b**) SPECT; (**c**) DCTWT; (**d**) Curvelet; (**e**) NSCT; (**f**) Liu-ASR; (**g**) Kim; (**h**) Zhu; (**i**) Proposed.

**Figure 10:**
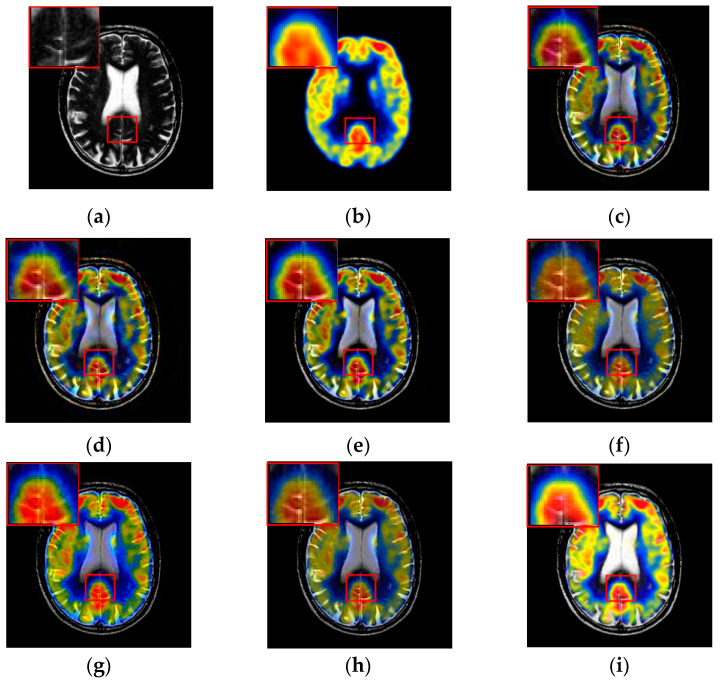
Fused results of MRI/PET medical images by different methods. (**a**) MRI; (**b**) PET; (**c**) DCTWT; (**d**) Curvelet; (**e**) NSCT; (**f**) Liu-ASR; (**g**) Kim; (**h**) Zhu; (**i**) Proposed.

To further demonstrate that the proposed method is equally effective for other types of medical image fusion, we test the other two categories of medical image fusion systems include MRI/SPECT and MRI/PET, and the fusion results are shown in Figure 9 and Figure 10. The details of fused images are zoomed in and presented in the bottom left corners of their respective images. 

With more careful observation, we can see that the spatial edge details and brightness information in Figure 9i and Figure 10i are more accurate compared with Figure 9c–h and Figure 10c–h generated by the six compared methods. This means the useful information from source images have been successfully transformed into the fused images, that is, our fused results have the best visual features. This is mainly because the novel construction of the overcomplete dictionary in this paper is composed of two parts include the brightness sub-dictionary and the edge detail sub-dictionary, which can fully express the significant features of the medical images. This will be very beneficial to the implementation of medical image fusion in practical medical assistance applications. In conclusion, the proposed algorithm has the best subjective visual effect.

### 3.4. Fusion Results by Objective Evaluation

To effectively evaluate the quality of fused images, we need take a quantitative evaluation for fused results, the metric can assess the perceptual quality of fused results consistently with visual subjective evaluation is highly desired. For medical image fusion problems, since the reference image (ground truth) does not exist in practice, quantitative evaluation of the quality of the fused image is not an effortless task. Over the last few decades, a number of fusions metrics have been proposed. Unfortunately, due to the various fusion scenarios, none of them are generally considered to be always more reasonable than other metrics. Therefore, it is often necessary to apply multiple indicators for comprehensive evaluation.

In this part, we employ five popular and representative quantitative indices to objectively evaluate all fusion results. These metrics include mutual information (MI) [43], edge information retention operator Q^AB/F^ [44], nonlinear correlation information entropy (Q_NCIE_) [45], image fusion metric-based on phase congruency (Q_P_) [46], and Piella’s Metric Q_S_ [47]. Among them, MI can reflect the degree of mutual information between the source images and the fused image; Q^AB/F^ can effectively evaluate the amount of the edge information transferred from the source images into the fused image; Q_NCIE_ is an information-based metric that measures the Correlation between fused image and the source images; Q_P_ indicates the degree of retaining significant saliency feature from the source images into the fused image. Q_S_ measures the contrast in the neighborhood of pixel.

For all of them, a larger value indicates better fusion results, and the codes of metrics include Q_NCIE_, Q_P_ and Q_S_ are implemented in an evaluation toolbox by Liu [48]. The quantitative evaluation results of all methods are presented in Table 2 and Table 3. By comparison, we can see that the Zhu’s method will marginally outperform our method for the Q_P_ evaluation value in MRI/PET images. But our method can obtain the best performance for other metrics, and the subjective visual effect of our result (Figure 10i) is slightly superior to it (Figure 10h). As a whole, Table 2 and Table 3 clearly indicate our method outperforms the other methods in terms of almost metrics.

Consideration of the subjective visual comparison and objective quantitative evaluation, one can finally conclude that our method can generate visually pleasant fused images contain abundant detail and brightness information in most cases and outperforms some competing methods on visual quality and objective evaluation.

### 3.5. Computational Efficiency Analysis

As previously mentioned, the proposed dictionary learning method can address the defects of traditional dictionary learning algorithms with some superfluous patches and low computation efficiency. In this subsection, the computational complexity of different fusion methods are analyzed, the results are shown in Table 4. It is well known that SR based fusion method are generally time consuming, especially for the case of learning a dictionary from source images. As can be seen from Table 4, we can see that the first three MST based methods are less time-consuming compared with other four SR based methods.

Nonetheless, compared with the three typical dictionary methods include Liu-ASR, Kim and Zhu methods, the proposed method saves more time. This demonstrates that our method could increase the computational efficiency more than some dictionary learning based fusion methods. Furthermore, the computational efficiency of our method still has much room for improvement, and it can be further improved if we fully optimize the speed of its implementation and by utilizing multithreading and performance boosters like Graphics Processing Unit (GPU) acceleration.

### 3.6. Extension to Other Type Image Fusion Issues

**Figure 11:**
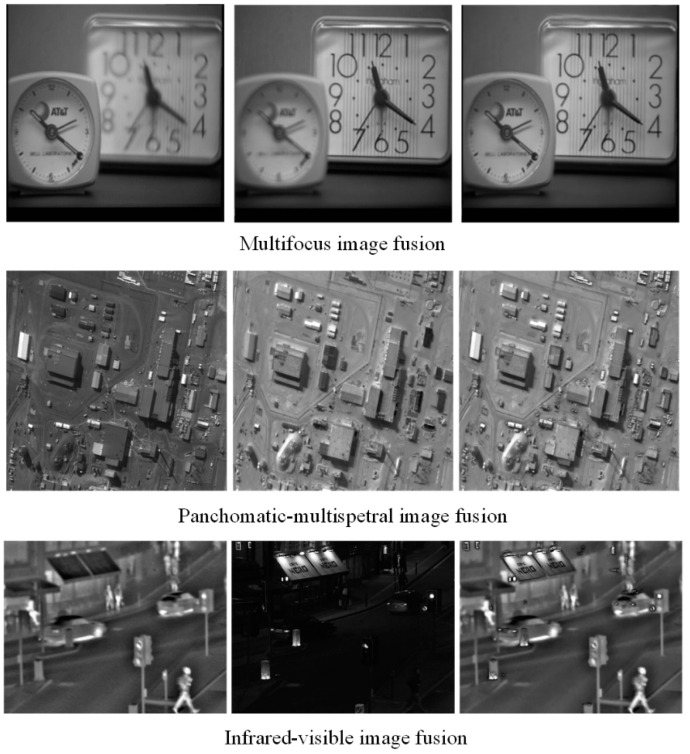
Other types of image fusion examples using the proposed method.

To exhibit the generalization ability of the proposed method, we extend its application to other types of image fusion including multifocus image fusion, panchromatic-multispectral image fusion and infrared-visible image fusion. The three fusion examples are shown in Figure 11, where the source images are displayed in the first two columns, and the fused results are listed in the last column. We can notice that the important feature include detail, sharp, edge and brightness feature et al. of source images are well preserved in the fused image. Among them, the fusion performance of infrared-visible image fusion is relatively high. This demonstrates that the proposed model can transfer useful information from the source images into the fused result [49]. Meanwhile, fewer undesirable artifacts are introduced in these fusion process, which indicate our method has strong robustness in these applications. Furthermore, compared with MRI and PET imaging, some new functional medical imaging modality, such as photoacoustic computed tomography (PACT) [50], resting-state functional connectivity (RSFC) and functional connectivity photoacoustic tomography (fcPAT) [51] etc., can offer better spatial resolutions with a fast, noninvasive and non-ionizing imaging modality to describe the brain physiology and pathology. Therefore, further study will be required to investigate them for image fusion in the near future.

## 4. Conclusions

SR based dictionary learning technology has been widely used in the medical image fusion field due to its superior performance. The core problem of these kinds of algorithms is constructing an informative and compact overcomplete dictionary with abundant information. Aiming at the traditional dictionary learning methods lack enough ability to express the source image information. This paper proposes a novel dictionary learning method based on brightness and detail clustering for medical image fusion. The proposed approach consists of three steps. Firstly, multi-layer ND filtering enhances the details of pre-training images, thus the weak information can be reinforced in the training set.

At the same time, we conduct the MSS on images to realize the multi-scale presentation of patches. Secondly, we propose SNE and MSF to classify the brightness and detail information patches groups, and then construct the brightness sub-dictionary and the detail sub-dictionary by K-SVD. The combination of the two sub-dictionaries can generate the final informative and compact dictionary. Finally, a SR model is established, and generates the encouraging fused results. Experimental analysis on some traditional as well the state-of-the art dictionary learning based medical fusion methods on four categories of medical images fusion shows that the proposed method has obvious superiority in both subjective visual effect and quantitative evaluation.

## Figures and Tables

**Table 1:** The number of patches by different size images.

	${\tilde{X}}_{l}$	${\tilde{X}}_{2,l}$	${\tilde{X}}_{3,l}$	${\tilde{X}}_{4,l}$	${\tilde{X}}_{5,l}$
Number of Patches	15625	3721	1600	841	529
Summation	15625	6691			

**Table 2:** The quantitative assessment of different methods for MRI/CT and MR-T1/MR-T2 fusion.

Images	Metric	Dctwt	Curvelet	NSCT	Liu-ASR	Kim	Zhu	Proposed
MRI/CT	MI	3.3878	3.1522	3.5582	3.7833	3.6642	3.6680	**4.9366**
	Q^AB/F^	0.5134	0.4581	0.5635	0.5404	0.4743	0.4620	**0.6254**
	Q_NICE_	0.8093	0.8086	0.8098	0.8105	0.8102	0.8102	**0.8161**
	Q_P_	0.3094	0.2460	0.3948	0.4353	0.3168	0.3469	**0.5421**
	Q_S_	0.7273	0.6881	0.7533	0.7720	0.7654	0.7631	**0.7996**
MR-T1/MR-T2	MI	2.9706	2.8620	3.0517	3.2929	3.1832	3.2811	**4.7856**
	Q^AB/F^	0.5395	0.4976	0.5613	0.5269	0.5008	0.4955	**0.6566**
	Q_NICE_	0.8074	0.8071	0.8076	0.8082	0.8079	0.8082	**0.8158**
	Q_P_	0.4513	0.3709	0.4693	0.4896	0.4089	0.4614	**0.6735**
	Q_S_	0.7290	0.6888	0.7659	0.8105	0.8042	0.8045	**0.8532**

The best scores of each metric are highlighted in bold faces.

**Table 3:** The quantitative assessment of different methods for MRI/SPECT and MRI/PET fusion.

Images	Metric	Dctwt	Curvelet	NSCT	Liu-ASR	Kim	Zhu	Proposed
MRI/SPECT	MI	2.7104	2.6430	2.7347	2.8251	2.7289	2.8235	**3.4636**
	Q^AB/F^	0.6646	0.6522	0.6635	0.6382	0.5712	0.6170	**0.6829**
	Q_NICE_	0.8063	0.8062	0.8064	0.8066	0.8063	0.8066	**0.8087**
	Q_P_	0.4283	0.3854	0.4540	0.5042	0.3123	0.4122	**0.5317**
	Q_S_	0.8890	0.8513	0.9097	0.9143	0.8950	0.9053	**0.9186**
MRI/PET	MI	2.4927	2.4660	2.5622	2.7089	2.5934	2.7501	**3.3282**
	Q^AB/F^	0.5208	0.5076	0.5585	0.5801	0.4746	0.5260	**0.6038**
	Q_NICE_	0.8055	0.8054	0.8057	0.8060	0.8057	0.8061	**0.8077**
	Q_P_	0.3198	0.2875	0.3366	0.4383	0.2661	**0.3641**	0.3519
	Q_S_	0.7642	0.7158	0.8296	0.8242	0.8017	0.8258	**0.8582**

The best scores of each metric are highlighted in bold faces.

**Table 4:** Time consumption of the different fusion methods (Time/s).

	Curvelet	DCTWT	NSCT	Liu-ASR	Kim	Zhu	Proposed
MRI/CT	16.15	7.89	25.42	86.66	61.67	55.54	35.24
MR-T1/MR-2	15.86	7.71	25.48	80.53	61.38	40.75	33.99
MRI/SPECT	39.27	14.11	75.76	173.29	55.20	226.69	88.84
MRI/PET	39.44	14.38	79.85	178.66	59.08	227.67	82.10

## References

[1] Du J., Li W., Lu K., Xiao B. (2016). An overview of multi-modal medical image fusion. Neurocomputing.

[2] Li H., He X., Tao D., Tang Y., Wang R. (2018). Joint medical image fusion, denoising and enhancement via discriminative low-rank sparse dictionaries learning. Pattern Recognit..

[3] Li S., Yang B., Hu J. (2011). Performance comparison of different multi-resolution transforms for image fusion. Inf. Fusion.

[4] Li H., Li X., Yu Z., Mao C. (2016). Multifocus image fusion by combining with mixed-order structure tensors and multiscale neighborhood. Inf. Sci..

[5] Goshtasby A., Nikolov S. (2007). Image fusion: Advances in the state of the art. Inf. Fusion.

[6] Du J., Li W., Xiao B., Nawaz Q. (2016). Medical image fusion by combining parallel features on multi-scale local extrema scheme. Knowl. Based Syst..

[7] Jiang Q., Jin X., Lee S., Yao S. (2017). A novel multi-focus image fusion method based on stationary wavelet transform and local features of fuzzy sets. IEEE Access.

[8] Lewis J., Callaghan R., Nikolov S., Bull D., Canagarajah N. (2007). Pixel and region based image fusion with complex wavelets. Inf. Fusion.

[9] Nencini F., Garzelli A., Baronti S., Alparone L. (2007). Remote sensing image fusion using the curvelet transform. Inf. Fusion.

[10] Do M.N., Vetterli M. (2005). The Contourlet Transform: An Efficient Directional Multiresolution Image Representation. IEEE Trans. Image Process..

[11] Li X., Li H., Yu Z., Kong Y. (2015). Multifocus image fusion scheme based on the multiscale curvature in nonsubsampled contourlet transform domain. Opt. Eng..

[12] Liu Y., Liu S., Wang Z. (2015). A general framework for image fusion based on multi-scale transform and sparse representation. Inf. Fusion.

[13] Palsson F., Sveinsson J.R., Ulfarsson M.O. (2017). Multispectral and Hyperspectral Image Fusion Using a 3-D-Convolutional Neural Network. IEEE Geosci. Remote Sens. Lett..

[14] Liu Y., Chen X., Wang Z., Wang Z.J., Ward R.K., Wang X. (2018). Deep learning for pixel-level image fusion: Recent advances and future prospects. Inf. Fusion.

[15] Li S., Kang X., Fang L., Hu J., Yin H. (2017). Pixel-level image fusion: A survey of the state of the art. Inf. Fusion.

[16] He C., Liu Q., Li H., Wang H. (2010). Multimodal medical image fusion based on IHS and PCA. Procedia Eng..

[17] Jiang Y., Wang M. (2014). Image fusion with morphological component analysis. Inf. Fusion.

[18] Zhang Q., Liu Y., Blum R., Han J., Tao D. (2018). Sparse representation based multi-sensor image fusion for multi-focus and multi-modality images: A review. Inf. Fusion.

[19] Shang L., Liu S., Zhou Y., Sun Z. (2017). Modified sparse representation based image super-resolution reconstruction method. Neurocomputing.

[20] Gu S., Zuo W., Xie Q., Meng D., Feng X., Zhang L. Convolutional Sparse Coding for Image Super-resolution. Proceedings of the IEEE International Conference on Computer Vision.

[21] Liu H., Liu Y., Sun F. (2015). Robust exemplar extraction using structured sparse coding. IEEE Trans. Neural Netw. Learn. Syst..

[22] Xu S., Yang X., Jiang S. (2017). A fast nonlocally centralized sparse representation algorithm for image denoising. Signal Process..

[23] Mourabit I., Rhabi M., Hakim A., Laghrib A., Moreau E. (2017). A new denoising model for multi-frame super-resolution image reconstruction. Signal Process..

[24] Karanam S., Li Y., Radke R. Person re-identification with discriminatively trained viewpoint invariant dictionaries. Proceedings of the IEEE International Conference on Computer Vision.

[25] An L., Chen X., Yang S., Bhanu B. (2016). Sparse representation matching for person re-identification. Inf. Sci..

[26] Bahrampour S., Nasrabadi N., Ray A., Jenkins W. (2015). Multimodal task-driven dictionary learning for image classification. IEEE Trans. Image Process..

[27] Li S., Yin H., Fang L. (2012). Group-sparse representation with dictionary learning for medical image denoising and fusion. IEEE Trans. Biomed. Eng..

[28] Li Y., Sun Y., Huang X., Qi G., Zheng M., Zhu Z. (2018). An Image Fusion Method Based on Sparse Representation and Sum Modified-Laplacian in NSCT Domain. Entropy.

[29] Wang K., Qi G., Zhu Z., Chai Y. (2017). A Novel Geometric Dictionary Construction Approach for Sparse Representation Based Image Fusion. Entropy.

[30] Yang B., Li S. (2010). Multifocus Image Fusion and Restoration with Sparse Representation. IEEE Trans. Instrum. Meas..

[31] Yang B., Li S. (2012). Pixel-level image fusion with simultaneous orthogonal matching pursuit. Inf. Fusion.

[32] Zhu Z., Chai Y., Yin H., Li Y., Liu Z. (2016). A novel dictionary learning approach for multi-modality medical image fusion. Neurocomputing.

[33] Kim M., Han D.K., Ko H. (2016). Joint patch clustering-based dictionary learning for multimodal image fusion. Inf. Fusion.

[34] Yin H. (2015). Sparse representation with learned multiscale dictionary for image fusion. Neurocomputing.

[35] Liu Y., Wang Z. (2015). Simultaneous image fusion and denoising with adaptive sparse representation. IET Image Process..

[36] Qi G., Wang J., Zhang Q., Zeng F., Zhu Z. (2017). An Integrated Dictionary-Learning Entropy-Based Medical Image Fusion Framework. Future Internet.

[37] Zhu Z., Yin H., Chai Y., Li Y., Qi G. (2018). A novel multi-modality image fusion method based on image decomposition and sparse representation. Inf. Sci..

[38] Pati Y.C., Rezaiifar R., Krishnaprasad P.S. Orthogonal matching pursuit: Recursive function approximation with applications to wavelet decomposition. Proceedings of the 27th Asilomar Conference on Signals, Systems and Computers.

[39] Li H., Liu X., Yu Z., Zhang Y. (2016). Performance improvement scheme of multifocus image fusion derived by difference images. Signal Process..

[40] Zhao H., Shang Z., Tang Y., Fan B. (2013). Multi-focus image fusion based on the neighbor distance. Pattern Recognit..

[41] Aharon M., Elad M., Bruckstein A. (2006). K-SVD: An Algorithm for Designing Overcomplete Dictionaries for Sparse Representation. IEEE Trans. Signal Process..

[42] Li H., Manjunath B.S., Mitra S.K. (1995). Multisensor image fusion using the wavelet transform. Graph. Models Image Process..

[43] Qu G., Zhang D., Yan P. (2002). Information measure for performance of image fusion. Electron. Lett..

[44] Xydeas C.S., Petrovic V. (2000). Objective image fusion performance measure. Electron. Lett..

[45] Wang Q., Shen Y., Jin J., Stathaki T. (2008). Performance Evaluation of Image Fusion Techniques. Image Fusion: Algorithms and Applications.

[46] Zhao J., Laganiere R., Liu Z. (2007). Performance assessment of combinative pixel-level image fusion based on an absolute feature measurement. IJICIC.

[47] Piella G., Heijmans H. A New Quality Metric for Image Fusion. Proceedings of the International Conference on Image Processing.

[48] Liu Z., Blasch E., Xue Z., Zhao J., Laganière R., Wu W. (2012). Objective assessment of multiresolution image fusion algorithms for context enhancement in night vision: A comparative study. IEEE Trans. Pattern Anal. Mach. Intell..

[49] Li H., Qiu H., Yu Z., Zhang Y. (2016). Infrared and visible image fusion scheme based on NSCT and low-level visual features. Infrared Phys. Technol..

[50] Zhao J., Xia J., Maslov K.I., Nasiriavanaki M., Tsytsarev V., Demchenko A.V., Wang L.V. (2013). Noninvasive photoacoustic computed tomography of mouse brain metabolism in vivo. NeuroImage.

[51] Nasiriavanaki M., Xia J., Wan H., Bauer A.Q., Culver J.P., Wang L.V. (2013). High-resolution photoacoustic tomography of resting-state functional connectivity in the mouse brain. Proc. Natl. Acad. Sci. USA.

